# Testing the effects of heterozygosity on growth rate plasticity in the seaweed *Gracilaria chilensis* (Rhodophyta)

**DOI:** 10.1002/ece3.4113

**Published:** 2018-05-07

**Authors:** Cristóbal F. Gallegos Sánchez, Jessica Beltrán, Verónica Flores, Alejandra V. González, Bernabé Santelices

**Affiliations:** ^1^ Departamento de Ecología Facultad de Ciencias Biológicas Pontificia Universidad Católica de Chile Santiago Chile; ^2^ Departamento de Ciencias Ecológicas Facultad de Ciencias Universidad de Chile Santiago Chile

**Keywords:** *Gracilaria chilensis*, growth rate plasticity, heterozygosity, phenotypic plasticity, ploidy

## Abstract

Heterozygosity has been positively associated with fitness and population survival. However, the relationship between heterozygosity and adaptive phenotypic plasticity (i.e., plasticity which results in fitness homeostasis or improvement in changing environments) is unclear and has been poorly explored in seaweeds. In this study, we explored this relationship in the clonal red seaweed, *Gracilaria chilensis* by conducting three growth rate plasticity experiments under contrasting salinity conditions and by measuring heterozygosity with five microsatellite DNA markers. Firstly, we compared growth rate plasticity between the haploid and diploid phases. Secondly, we compared growth rate plasticity between diploids with different numbers of heterozygous loci. Finally, we compared growth rate plasticity between diploid plants from two populations that are expected to exhibit significant differences in heterozygosity. We found that, (i) diploids displayed a higher growth rate and lower growth rate plasticity than haploids, (ii) diploids with a higher number of heterozygous loci displayed lower growth rate plasticity than those exhibiting less heterozygosity, and (iii) diploid sporophytes from the population with higher heterozygosity displayed lower growth rate plasticity than those with lower heterozygosity. Accordingly, this study suggests that heterozygosity is inversely related to growth rate plasticity in *G. chilensis*. However, better genetic tools in seaweeds are required for a more definitive conclusion on the relationship between heterozygosity and phenotypic plasticity.

## 1 INTRODUCTION

Genetic diversity is usually considered necessary for population survival and adaptability in changing environments (Booy, Hendriks, Smulders, Van Groenendael, & Vosman, 2000). One of the most common measurements of genetic diversity is heterozygosity, which represents the combinations of alleles within organisms and is independent of allelic or genotypic richness at the population level. The level of heterozygosity seems to have a profound effect on organism performance. For example, a positive correlation between heterozygosity and fitness components (e.g., growth rate, survival, or reproduction) has been found for a variety of organisms (see Reed & Frankham, 2003), whereas low heterozygosity has been associated with inbreeding depression and reductions in fitness (e.g., Allendorf & Leary, 1986; Crnokrak & Roff, 1999; Eldridge et al., 1999; Saccheri, Brakefield, & Nichols, 1996).

Heterozygosity is to some extent related to ploidy. Haploid organisms have only one allele per locus (i.e., they are hemizygous); hence, they cannot display any degree of heterozygosity. In contrast, diploid organisms could potentially show some degree of heterozygosity (and usually do), which is expected to increase with greater levels of ploidy (Leary, Allendorf, Knudsen, & Thorgaard, 1985). In this context, several authors have suggested that the positive correlation sometimes found between ploidy and growth rate (e.g., Guillemin, Sepúlveda, Correa, & Destombe, 2013; Mendoza & Haynes, 1974; Petit et al. 1996), and between ploidy and stress resistance (Van Laere et al., 2011) is related to heterosis.

Additionally, heterozygosity has been positively associated with adaptive phenotypic plasticity (Mitton & Grant, 1984). Phenotypic plasticity is defined as the change in the phenotype of a single genotype in different environments (Schmaulsen, 1949) and is usually described as a reaction norm (Schlichting & Pigliucci, 1995). When adaptive, plasticity allows organisms to maintain high constant fitness across environments through the modification of one or more traits (i.e., low fitness plasticity). Therefore, it relates to broader environmental tolerance and is important for organisms living in changing environments. Nevertheless, the relationship between heterozygosity and plasticity is far more contentious than its relationship to fitness in a single environment, and there seems to be no general trend (Booy et al., 2000). Some studies have shown that organisms with higher heterozygosity are less sensitive to environmental stress and display lower fitness plasticity (Samollow & Soulé, 1983; Yampolsky & Kalabushkin, 1991). However, other studies have failed to find such a relationship (Schlichting & Levin, 1984; Wetzel, Stewart, & Westneat, 2012; Wu, 1997) and Schlichting (1986) even argued that there was no reason to justify it. Thus, the effect of heterozygosity in the plasticity of fitness components is not entirely clear, even less so in seaweeds.

**Figure 1:**
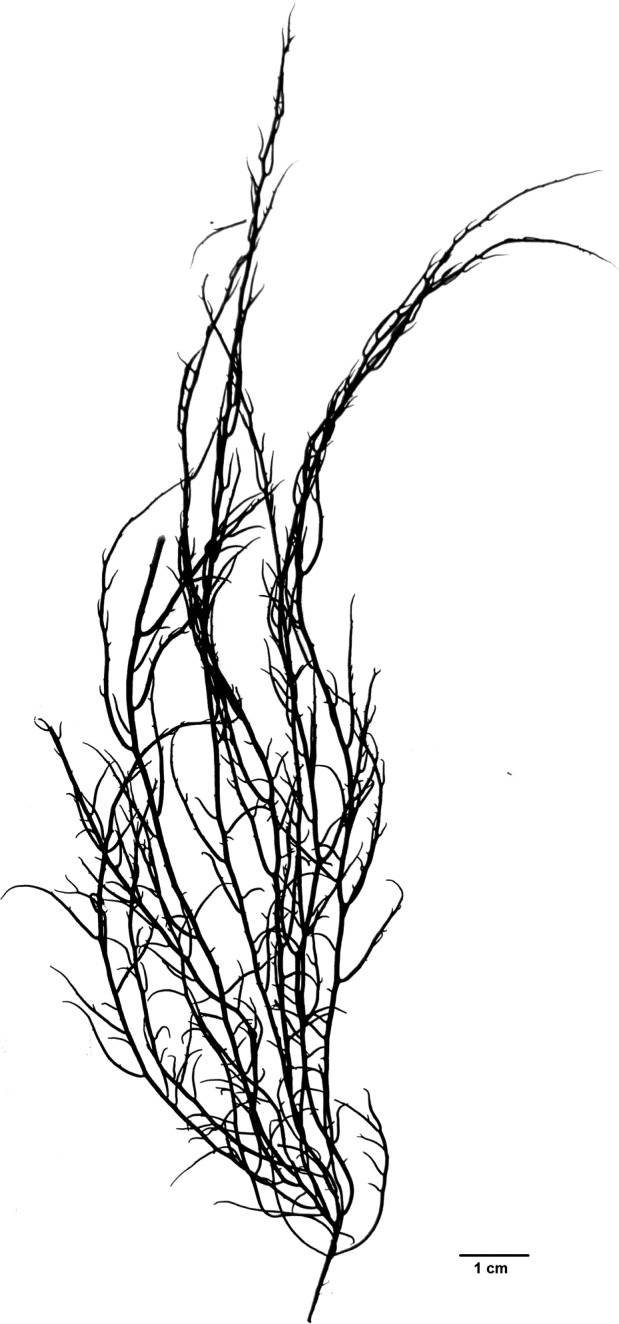
A photograph showing the portion of a genet (a ramet) of *Gracilaria chilensis* equivalent to the ones used in the experiment 3 of this study (sporophyte, 2n). Notice that due to the modular and clonal nature of this species, any new fragment derived from this ramet can potentially give birth to a new organism

The red seaweed *Gracilaria chilensis* (Rhodophyta) occurs in estuarine habitats, where salinity is highly variable. This is a modular and branched seaweed (Figure 1) that can grow attached to rocks, buried in the sand or free living, either in the intertidal or subtidal zone (Bird, McLachlan, & Oliveira, 1986). Some populations of *G. chilensis* have been farmed in Chile by clonal propagation (Santelices & Doty, 1989); it has been shown that farmed populations exhibit a reduction in genotypic diversity, but an increase in observed heterozygosity (Guillemin et al., 2008), which is likely due to the accumulation of mutations as a result of clonality and the absence of sexual reproduction (Balloux, Lehmann, & Meeûs, 2003; Bengtsson, 2003).

The study by Guillemin et al. (2008) is one of the first to uncover genetic patterns in *G. chilensis* populations. Even though they have not tested the ecological effects of their findings, they do discuss and recognize the importance of heterozygosity in terms of its causes and consequences for fitness. Krueger‐Hadfield et al. (2016) also found an increase in heterozygosity in introduced populations of *G. vermiculophylla*, which they suggested was related to colonization success. Nevertheless, it is unclear whether this increase in heterozygosity in *Gracilaria* spp. could be adaptive or not. Experimental studies of this kind are scarce in macroalgae, probably due to the limited genetic tools available and because they have mostly been used for population genetics research (Valero, Engel, Billot, Kloareg, & Destombe, 2001).

Considering the perceived importance of heterozygosity to organism performance, we explored the relationship between heterozygosity—using five microsatellite DNA markers—and growth rate plasticity in the clonal red seaweed, *G. chilensis,* cultured under different salinity conditions. To this end, the ideal would be to construct strictly heterozygous and homozygous strains in the laboratory and then compare their response. However, because of the technical difficulties of this task, three different experiments were designed as first approximations to this problem. First, we evaluated whether the diploid phase of *G. chilensis* shows lower growth rate plasticity than the haploid phase, considering their differences in ploidy may in part relate to heterozygosity. In the second experiment, we evaluated whether different levels of heterozygosity in diploid and individual genotypes (sporophytes) were related to differences in growth rate plasticity. Lastly, we tested whether different levels of heterozygosity between two *G. chilensis* populations were related to differences in growth rate plasticity (i.e., a population level response). The results are discussed in terms of the controversial relationship between heterozygosity and phenotypic plasticity and their conjunct potential role in population survival under changing environments.

## 2 MATERIALS AND METHODS

### 2.1 Experiment 1: ploidy versus growth rate plasticity

#### 2.1.1 Sampling design

A total of 50 samples of *G. chilensis* were randomly collected from a wild, sexually reproducing population in Maullín, Chile (41° 37′S, 73° 35′O), with a minimum distance of 1 meter from each other to avoid re‐sampling the same genet. Samples were then transported in sealed plastic bags placed inside a cooler at 5°C to the laboratory in Santiago. Among these, 23 sporophytes (diploids) and 23 female gametophytes (haploids) were identified in the laboratory by the presence of reproductive structures. From each genet, two adjacent apices (clonal ramets) of ~6 cm were isolated to measure the genet plasticity (Monro & Poore, 2004). This methodology reduces genetic variation between individuals to the minimum possible, which is necessary for a more accurate estimate of plasticity (Valladares, Sanchez‐Gomez, & Zavala, 2006). Only vegetative ramets were identified and selected by a thorough microscopic inspection. Also, there was no visual sign indicating that the selected ramets could have been the result of in situ gametophyte germination as is possible in *Gracilaria* spp. (e.g., Destombe, Valero, Vernet, & Couvet, 1989; Kain and Destombe 1995; Polifrone, De Masi, & Gargiulo, 2006).

#### 2.1.2 Experimental procedures

The basal portion of each ramet (~3 cm) was cut for genetic analysis, while the apical portion (~3 cm) was cultured. Both clonal apices corresponding to each plant (*n* = 23) and ploidy level were randomly assigned and cultured under 10‰ and 25‰ of salinity for 30 days in 100 ml flasks with 75 ml of SFC culture medium (Correa & McLachlan, 1991) under standard laboratory conditions (13 ± 2°C, 30 ± 5 μmol photons m^−2^ s^−1^, 12:12 light:dark). We selected salinity as a factor as *G. chilensis* occurs in estuarine habitats; then, adaptation to salinity variation is especially important in this species. In the same way, the levels of salinity selected in our experiment represent the natural salinity variation experimented by *G. chilensis* in the wild, which grows under 8–30‰ of salinities, with maximum growth at approximately 23‰ (Bird et al., 1986).

The culture medium was changed three times a week, and salinity levels were achieved by diluting seawater and then adding the nutrients corresponding to SFC medium. After being blot dried, each apex was weighted at the beginning and at the end of the experiment (30 days) with an analytical balance (Precisa Instruments AG, model 290 TYP, Sweden) to measure its wet weight. This was then used to estimate the growth rate as the increase in biomass over time.

##### Genetic characterization and heterozygosity

Because this was assessed solely to corroborate the (ha)ploidy of gametophytes and the presence of heterozygosity in sporophytes, from the total of 23 genets per phase only 12 randomly chosen genets were genotyped (24 samples in total). To this end, five microsatellite loci (7F12, 8B2, 7D3, 6C7, 2B2) previously developed for this species were used (Guillemin, Destombe, Faugeron, Correa, & Valero, 2005). These markers offer an opportunity to investigate preliminary trends in the relationship between ecological responses and genetic diversity in a taxon in which this has not been explored previously. We recognize that there are limitations to the use of just a few microsatellite markers and this is discussed later.

The samples were first dried in silica gel for 2 weeks. Afterward, DNA extraction was carried out by grinding the tissue samples and using a slightly modified version of the CTAB extraction protocol modified for plants containing high polysaccharide and polyphenol components (Porebski, Bailey, & Baum, 1997). Following Guillemin et al. (2005), PCR amplifications were carried out in 10 μl of reaction volume with 2 μl of template DNA (20 ng/μl), 5 μl (2X) of GoTaq Green Master Mix (Promega), 0.25 μl (40 ng/μl) of each primer DNA (forward and reverse), 1 μl (10 mg/ml) of bovine serum albumin (BSA), 1.2 μl (25 mM) of MgCl_2_, 0.25 μl (40 ng/μl) of M13 (FAM, VIC, NED or PET) fluorescently labeled tail (Schuelke, 2000), and the remaining volume filled with nuclease‐free water. PCR reactions were performed using a thermocycler (Applied Biosystems, GeneAmp PCR System 9700, USA), whereas PCR products were then separated and scored by capillary electrophoresis using a genetic analyzer (Applied Biosystems, model 3130XL, USA). This was followed by scoring allele size using the Peak Scanner Software v1.0 (Applied Biosystems). All nonamplified or unclear samples were re‐amplified and re‐scored to avoid missing data. Two independent observers recorded all results manually. Fragment sizes were transferred to Microsoft Excel for further analysis. Observed heterozygosity was calculated using the GenAlEx 6.5 software (Peakall & Smouse, 2006).

##### Growth rate and plasticity of this trait

Biomass data at the beginning and at the end of the experiment (30 days) were used to calculate the specific growth rate (SGR; Hurd, Harrison, Bischop, & Lobban, 2014; Troell et al., 1997) using the following equation: % SGR = 100*(In (t_30_/t_0_)/30), in which t_0_ and t_30_ correspond to the biomass (in grams) measured at the beginning and after 30 days of culture, respectively. A two‐way ANOVA was performed to test the effects of ploidy, salinity, and of the interaction of both on SGR. A significant effect of the interaction term reveals whether there are significant differences in the slope of reaction norms and therefore in growth rate plasticity between phases.

### 2.2 Experiment 2: number of heterozygous loci versus growth rate plasticity

#### 2.2.1 Sampling design

A total of 15 samples of *G. chilensis* were randomly collected from a wild population in Maullín, Chile (41° 37′S, 73° 35′O), following the methods described above. They were all identified as sporophytes due to the presence of tetrasporangia (reproductive structures) by microscopic inspection. As above, two adjacent, nonmature apices (clonal ramets) of ~6 cm were isolated to measure genet plasticity (Monro & Poore, 2004).

#### 2.2.2 Experimental procedures

The lower portion of the 15 apices (~3 cm) was cut and used for genetic analysis while the upper portion (~3 cm) was randomly assigned and cultured under 10‰ and 25‰ of salinity for 30 days in 100 ml flasks with 75 ml of SFC culture medium (Correa & McLachlan, 1991) under standard laboratory conditions (13 ± 2°C, 30 ± 5 μmol photons m^−2^ s^−1^, 12:12 light:dark). The culture medium was changed three times a week, and salinity levels were achieved as previously described. Following the procedures of the first experiment, each apex was wet weighted twice to calculate its growth rate.

##### Heterozygosity

One apex from each of the 15 clonal pairs was randomly chosen and genotyped following the methods described above and using the same five microsatellite loci. Considering the neutrality of microsatellite genetic markers, observing a different response between homozygotes and heterozygotes on a locus‐by‐locus basis is unlikely. Therefore, a multilocus approach was adopted by counting the total number of heterozygous loci (NHL) for each sporophyte and then classifying them according to this criterion. This was used as a measure of the heterozygosity level of each individual genotype (Shikano & Taniguchi, 2002). Hence, the level of heterozygosity was considered a discrete variable with six possible values in this case (0, 1, 2, 3, 4, or 5 heterozygous loci).

##### Growth rate and plasticity of this trait

The specific growth rate (SGR) was calculated for each ramet following the procedures described above. A two‐way ANOVA was performed to test the effect of NHL, salinity and of the interaction of both on SGR. A significant effect of the interaction term reveals whether there are significant differences in the slope of the reaction norms and therefore in growth rate plasticity between heterozygosity levels.

### 2.3 Experiment 3: heterozygosity versus growth rate plasticity comparing two populations

#### 2.3.1 Sampling design

Samples of *G. chilensis* were collected from a wild population in Maullín (41° 37′ S, 73° 35′ W) and from a farmed population in Piedra Azul (41° 30′ S, 72° 47′ W). As shown by a previous study (Guillemin et al., 2008), these populations ought to show differences in observed heterozygosity, which should be higher for the farmed one. Both sites are estuarine habitats in the Los Lagos Region, near the city of Puerto Montt, Chile, and show similar salinity variation ranging from 10‰ to 35‰ (Huanel, 2011; Westermeier, Gómez, & Rivera, 1993). Approximately 60 genets were sampled from each population initially, discarding in situ mature gametophytes. According to Guillemin et al. (2008), the farmed population displays an absence of gametophytes and there is a skew toward sporophytes in the wild population. The initial 60 genets were examined under the microscope and mature sporophytes were discarded; thus, 50 vegetative genets were selected from each population for the experiment. A subset of 20 genets per population were genotyped, from which a 100% were identified as sporophytes (diploids) by the presence of at least one heterozygous locus. Therefore, this methodology let us safely assume all the experimental material corresponded to vegetative sporophytes.

#### 2.3.2 Experimental procedures

Each genet (50 per population) was divided into three equivalent clonal clumps such as the one in Figure 1 (i.e., three ramets corresponding to 1/3 of the original genet, with similar size and weight of 2 ± 0.2 g), which were kept independently, thus forming three sets of experimental clonal populations for each population type (farmed and wild) with 50 individuals each. Because of limited resources, a tissue sample was taken comprising only 20 randomly chosen plants from each of the six experimental populations to be genotyped and to calculate their observed heterozygosity. The clonal clumps were then cultivated at three different salinities (10‰, 25‰, and 40‰) in 200 ml flasks with 150 ml of SFC culture medium (Correa & McLachlan, 1991) under standard laboratory conditions (13 ± 2°C, 42 ± 5 μmol photons m^−2^ s^−1^, 12:12 light:dark) for 30 days. The upper salinity level was included in the experiment to explore differences in growth rate plasticity over a broader salinity range and to have a minimum of three experimental populations by population type. It was chosen to maintain the same difference in salinity between levels (i.e., 15‰). The culture medium was changed three times a week, and salinity levels were achieved by diluting seawater or adding NaCl as appropriate and then adding the nutrients corresponding to SFC medium. Following the procedures of the previous experiments, each clump was weighted at the beginning and after 30 days of culture to calculate its growth rate.

##### Genetic analysis and heterozygosity

Of the 50 genets from each experimental population, only 20 were sampled to be genotyped using the methods described above and then calculate their heterozygosity. Clonal propagation in *G. chilensis* was expected, especially in the farmed population (Guillemin et al., 2008). Therefore, two identical multi‐locus genotypes (MLGs) may be the result of sampling the same genet twice or sampling different genets (originated from two distinct sexual reproduction events) that share exactly the same alleles at all loci (Arnaud‐Haond et al., 2005). In the first, and perhaps the more common scenario, results could be altered by an overrepresentation of a few genets in the sample, which is more likely in the farmed population. Consequently, MLGs were identified and a correction analysis was performed using the software GeneClone 2.0 (Arnaud‐Haond & Belkhir, 2007) in which repeatedly sampled genets can be identified and eliminated from the dataset and subsequent analyses. The latter is performed by estimating *p*_*sex*_, the probability for a given MLG to be observed in *N* samples as a consequence of different sexual reproduction events (Tibayrenc, Kjellberg, & Ayala, 1990). Following Guillemin et al. (2008), when *p*_*sex*_ was lower or equal to 0.05, identical MLGs were considered the same genet and only one of them kept in the dataset. When *p*_*sex*_ was greater than 0.05, identical MLGs were considered different genets and all of them used for further analyses. This analysis was performed independently in the six experimental populations (i.e., three sets of the farmed population and three sets of the wild population, with 20 genotyped samples each). Observed heterozygosity was then calculated in each experimental population using the GenAlEx 6.5 software (Peakall & Smouse, 2006) and averaged by population type (*n* = 3). Statistical differences in observed heterozygosity by population were evaluated using an ANOVA test, after conducting tests for normality and homoscedasticity.

##### Growth rate and plasticity of this trait

The specific growth rate (SGR) was calculated for each clump following the procedures described in the previous experiments. In this case, the data were highly variable, especially at the lower salinity level. Hence, SGR data were divided into two separate analyses. First, the number of dying (negative SGR) vs. the number of growing clumps for each experimental population was counted and differences between populations were evaluated using a G‐test of independence (Schmitt, Hay, & Lindquist, 1995). Furthermore, a two‐way ANOVA was performed on SGR considering only growing clumps (those with positive SGR), and using population and salinity as factors. The interaction component in the ANOVA indicates variation in growth rate plasticity among populations. Because the overrepresentation of a few resampled genets may alter the effects of population and therefore heterozygosity on growth rate plasticity, the above analysis was repeated using only genotyped samples and removing repeated genets. In all cases, data were previously tested for homoscedasticity and normality, and both assumptions were met. All the previously described statistical analyses were performed using the R Studio software (v0.99.903; RStudio, Boston, USA).

## 3 RESULTS

### 3.1 Experiment 1: ploidy and growth rate plasticity

#### 3.1.1 Genetic characterization and heterozygosity

As expected, only one allele was found in all haploid samples, thus confirming their (ha)ploidy. On the other hand, diploids presented a value of observed heterozygosity (H_O_) of 0.45.

#### 3.1.2 Growth rate and plasticity of this trait

**Figure 2:**
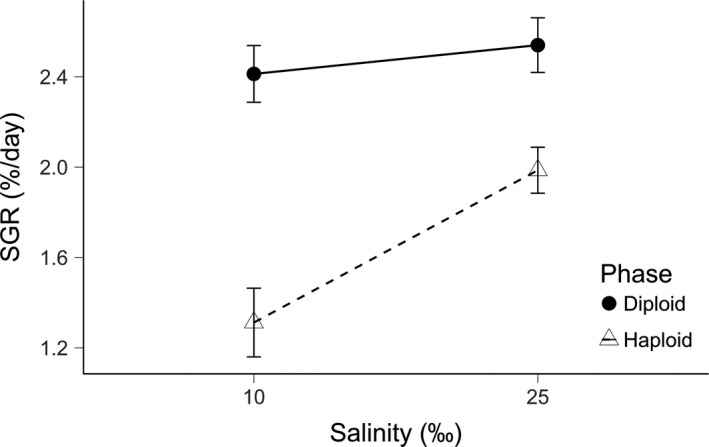
Mean specific growth rate (SGR) in diploid (sporophytes) and haploid (gametophytes) thalli of *Gracilaria chilensis* from Maullín, Chile in each salinity level after 30 days of culture. Lines connecting the dots represent SGR mean reaction norms for each ploidy level. Error bars show standard error (*n* = 23)

Diploids presented a higher mean SGR in both salinities (10 and 25‰) compared to haploids. Additionally, the former displayed similar SGR among salinity levels, while the latter displayed a decrease in SGR in 10‰ of salinity compared to 25‰ (Figure 2). There was a significant effect of ploidy on SGR (*F*_0.05 (1, 90)_ = 42.92, *p *<* *.0001), of salinity on SGR (*F*_0.05 (1, 90)_ *= *9.79, *p *<* *.01), and of the interaction between them (*F*_0.05 (1, 90)_ *= *4.69, *p *<* *.05). This suggests that each phase responds differently to salinity and that there are statistically significant differences between both reaction norms and consequently, their growth rate plasticity.

### 3.2 Experiment 2: number of heterozygous loci versus growth rate plasticity

#### 3.2.1 Heterozygosity

In terms of the level of heterozygosity for each individual thallus, from the 15 pairs of clones used in this experiment, seven (46.7%) presented three heterozygous loci and eight (53.3%) presented two heterozygous loci. These were the only two levels of heterozygosity found among the genotyped thalli.

#### 3.2.2 Growth rate and plasticity of this trait

**Figure 3:**
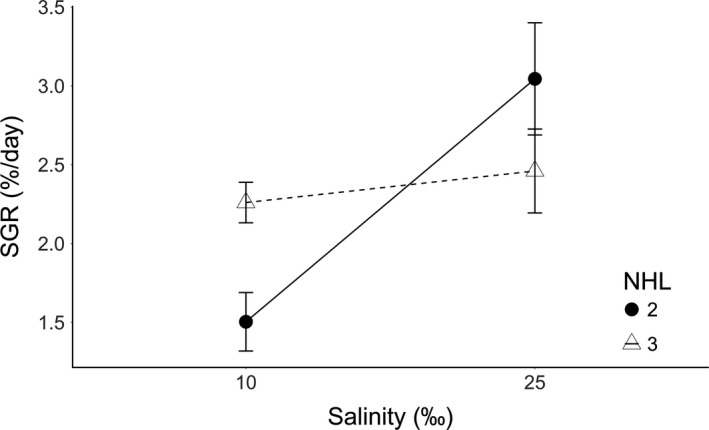
Mean specific growth rate (SGR) in sporophytes of *Gracilaria chilensis* with three and two heterozygous loci (NHL) from Maullín, Chile in each salinity level after 30 days of culture. Lines connecting the dots represent SGR mean reaction norms for each level of heterozygosity. Error bars show standard error (*n* = 7 for NHL = 3 and *n* = 8 for NHL = 2)

Thalli with three heterozygous loci presented a similar mean SGR at both salinity levels. However, thalli with only two heterozygous loci showed a decrease in SGR at the lower salinity compared to 25‰ (Figure 3). The number of heterozygous loci alone had no effect on SGR (*F*_0.05 (1, 26)_ = 0.11, *p *=* *.739), but the effect of salinity on SGR was significant (*F*_0.05 (1, 26)_ = 12.87, *p *<* *.002), as well as the interaction of both factors (*F*_0.05 (1, 26)_ = 6.88, *p *<* *.02), indicating differences in the slope of the reaction norms and in growth rate plasticity among sporophytes with two or three heterozygous loci.

### 3.3 Experiment 3: heterozygosity versus growth rate plasticity comparing two populations

#### 3.3.1 Genetic analysis and heterozygosity

Genetic analysis revealed a lower number of different MLGs and of different genets in the farmed experimental populations compared to the wild ones (Table 1). Only different genets were kept in the dataset and used to estimate the observed heterozygosity, which was averaged by type of population per origin (farmed or wild). As expected, observed heterozygosity (H_O_) was higher for the farmed experimental populations (0.620 ± 0.047) (mean ± se) than for the wild ones (0.397 ± 0.024), and this difference was statistically significant (*F*_0.05 (1, 4)_ = 17.54, *p *<* *.02).

**Table 1:** Number of different multi‐locus genotypes (MLGs) and number of different genets identified in each experimental population by estimating *P*_sex_ probability using the GenClone 2.0 software (see text for references and further details)

Experimental population	Number of genotyped samples	Number of different MLGs	Number of different genets
Wild—S10	20	19	19
Wild—S25	20	20	20
Wild—S40	20	20	20
Farmed—S10	20	9	12
Farmed—S25	20	8	11
Farmed—S40	20	9	12

#### 3.3.2 Growth rate and plasticity of this trait

After 30 days of culture under different salinity treatments, the farmed experimental populations (with higher heterozygosity) showed overall a higher percentage of growing plants (positive SGR) compared to the wild ones, and both types of populations were mostly affected by the lowest salinity level (Table 2). At a stressful salinity condition of 10‰, only 22% of plants from the wild population exhibited growth (with positive SGR), whereas the farmed population showed a significantly higher rate (42%, *G* = 4.65, *p *=* *.05).

**Table 2:** Number and percentage of growing plants by population and salinity level after 30 days of culture under experimental conditions

Population	Salinity (‰)	Status		% Growing
		Dying	Growing	
Wild	10	39	11	22
	25	5	45	90
	40	3	47	94
	Total	47	103	68.7
Farmed	10	29	21	42
	25	0	50	100
	40	1	49	98
	Total	30	120	80

**Figure 4:**
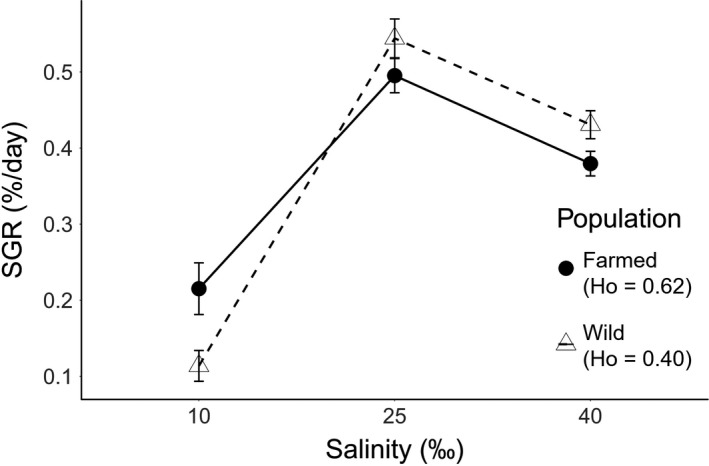
Mean specific growth rate (SGR) for each experimental population of *Gracilaria chilensis* by population type (farmed: Piedra Azul and wild: Maullín) in each salinity level after 30 days of culture. Connecting lines represent SGR mean reaction norms. Error bars show standard error (*n*‐values can be found in Table 2, number of growing plants)

Considering only growing plants, in terms of the mean SGR, a similar pattern was found to the one just described. The lowest mean SGR for both populations was found at 10 ‰ of salinity, and the wild population showed a lower SGR compared to the farmed one. Both populations presented maximum SGR at 25 ‰ of salinity (optimal condition) and a decrease at 40‰ (Figure 4). Population type alone had no effect on SGR (*F*_0.05 (1, 217)_ = 0.00, *p *=* *.98), but the effect of salinity was significant (*F*_0.05 (2, 217)_ = 70.04, *p *<* *.0001) as well as the interaction between both factors (*F*_0.05 (2, 217)_ = 3.54, *p *<* *.05). The significance of the interaction term indicates that there were significant differences in reaction norm slopes among populations and in growth rate plasticity. Figure 4 shows that this difference arose from the lower salinity range (i.e., 10‰ to 25‰), with no differences in the reaction norm slopes observed in the upper salinity range between populations.

**Figure 5:**
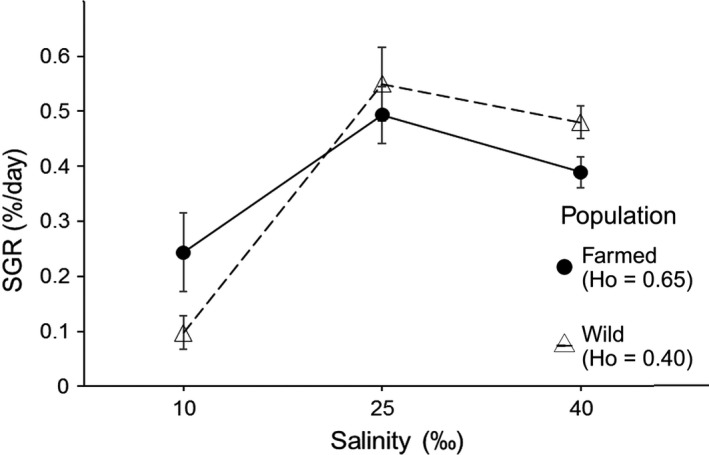
As in Figure 4, mean SGR for each experimental population of *Gracilaria chilensis* grown under each salinity level. In this case, only genotyped samples were used and repeated genets were removed from the analysis; however, a similar trend can be observed in Figure 4. Error bars show standard error and *n*‐values are as follows: W10 = 6, W25 = 19, W40 = 20, F10 = 4, F25 = 11, F40 = 11, where W stands for wild population, F for farmed population, and 10, 25, and 40 represent the salinity levels in which samples where grown

After comparing growth rate plasticity between populations, while only considering genotyped samples and eliminating repeated genets from the analysis, a similar result was observed in qualitative terms (Figure 5). As above, there was no significant effect of population (*F*_0.05 (1, 65)_ = 1.85, *p *=* *.179), but there was a significant effect of salinity on growth rate (*F*_0.05 (2, 65)_ = 25.93, *p *<* *.001). As can be observed in Figure 5, there were differences in reaction norm slopes between populations in the lower salinity range; however, as per de ANOVA, no significant differences were observed this time (*F*_0.05 (2, 65)_ = 2.56, *p *=* *.085).

## 4 DISCUSSION

Even though the positive effect of heterozygosity on fitness in single environments is more widely accepted (Booy et al., 2000), the relationship between the former with the plasticity of fitness traits across environments is controversial and not always recognized (Schlichting, 1986). Here, we performed three experiments as an initial and pioneer step to attempt to explore this relationship in macroalgae. We acknowledge that the low number of microsatellites used in this study limits the strength of the ensuing conclusions (Selkoe & Toonen, 2006). However, genetic tools in seaweeds are scarce and even more in *G. chilensis*. Until now, these have been the only microsatellites developed for this species, which calls attention to the need for further development in this area. Nevertheless, the combination of results gathered in this research seems to support the idea that more heterozygous individuals of *G. chilensis* have higher fitness and are also better able to maintain fitness in changing salinity environments. These two consequences combined (i.e., higher fitness in single environments and higher fitness maintenance across environments), could be and adaptive response and suggests that individuals with higher heterozygosity could have higher levels of phenotypic plasticity.

### 4.1 Ploidy and growth rate plasticity

The genetic characterization confirmed the samples ploidy, with hemizygous gametophytes of *G. chilensis* and with diploid sporophytes displaying a similar level of heterozygosity than what had previously been reported in the sampled population (Maullín; Guillemin et al., 2008). In terms of performance, we observed significant differences in growth rate and growth rate plasticity between both phases of the *G. chilensis* life cycle, in which diploids were able to maintain higher growth rates among the evaluated salinity conditions with consequently lower growth rate plasticity. Haploids instead displayed lower growth rates and a higher growth rate plasticity.

A higher growth rate of diploid versus haploid individuals was already found by a previous study of the same species (Guillemin et al., 2013) as well as for other seaweed species (González & Meneses, 1996; Zuccarello, Yeates, Wright, & Bartlett, 2001). In land plants, some studies have evidenced a positive effect of ploidy level on stress resistance in relation to phenotypic plasticity (Scholes & Paige, 2015; Van Laere et al., 2011), as well as on invasiveness capacity (Pandit, Pocock, & Kunin, 2011). However, to our knowledge, this is the first report of differences in adaptive plasticity between life phases in seaweeds.

Nevertheless, ploidy and heterozygosity may not be the only difference between *G. chilensis* life cycle phases. Despite them being isomorphic, it is currently accepted that subtle yet important morphological and physiological differences between the life phases of isomorphic seaweeds may exist (Thornber, Stachowicz, & Gaines, 2006). For example, differences in the chemical structure of cell walls between both phases of some *Gracilaria* species have been described (Lahaye, Rochas, & Yaphe, 1984). In *Ectocarpus siliculosus*, Thomas and Kirst (1991) showed that diploid sporophytes exhibited a higher tolerance to salt stress than haploid gametophytes; however, no tolerance improvement was observed in higher ploidy strains (3n and 4n). This might suggest that the higher tolerance of diploids compared to haploids is an intrinsic property of sporophytes rather than a consequence of higher heterozygosity in the first. Therefore, how far‐reaching the phenotypic consequences of ploidy are and the extent to which the results presented here relate to heterosis remains unclear and requires further research. However, considering the positive effects that heterozygosity can have on fitness (Reed & Frankham, 2003) and the results from the other experiments presented here, there are reasons to suspect they may be significant.

### 4.2 Number of heterozygous loci and growth rate plasticity

Comparing diploid individuals, we found that the sampled sporophytes showed only two levels of heterozygosity: two and three heterozygous loci. Consequently, it was not possible to evaluate continuity in the response, in which we would have expected growth rate plasticity to consistently decrease with the presence of more heterozygous loci. Despite this, we found that *G. chilensis* sporophytes (2n) with three heterozygous loci were less sensitive to low salinity (stressful condition) and displayed lower growth rate plasticity than those with only two heterozygous loci, supporting the role of heterozygosity in growth rate maintenance. This observed difference in growth rate plasticity is noteworthy as it was produced by a difference of only one locus. However, these results require further investigation, especially with the use of a greater number of microsatellites, which would allow exploration of a correlation between the number of heterozygous loci present in each genotype and its growth rate plasticity. Nevertheless, the results do point to an effect of heterozygosity on growth rate plasticity in *G. chilensis* which warrants further investigation.

### 4.3 Heterozygosity and growth rate plasticity comparing two populations

The genetic comparison between diploids from different populations of *G. chilensis* showed the same pattern as the one described by Guillemin et al. (2008), with lower genotypic richness and higher heterozygosity in farmed rather than wild populations. In addition, our study evidenced that the farmed population showed a significantly greater number of growing plants (i.e., with positive SGR) than those of the wild population, suggesting that *G. chilensis* from the population with highest heterozygosity (farmed) may be less sensitive to salt stress and more resistant to these conditions. The latter is probably due to a previous selective process carried out by farmers, which may have increased both heterozygosity and the tolerance of *G. chilensis* thalli.

In terms of growth rate response, even though we did not detect any population effect on growth rate, we did find a significant effect of the interaction between population and salinity, evidencing significant differences between both reaction norms. As both populations experience similar salinity ranges in their habitat (Huanel, 2011; Westermeier et al., 1993), these results are unlikely to be triggered by local adaptation to their respective environments. Therefore, in light of the results discussed above, the difference in growth rate plasticity between both populations is likely to occur due to the different levels of observed heterozygosity they displayed.

As explained in the methodology, differences in growth rate plasticity between populations were assessed a second time using only genotyped samples and eliminating repeated genets from the analysis. In qualitative terms, the results remain similar (Figures 4 and 5) which suggests consistency. However, no significant difference in growth rate plasticity was observed between populations this time (*p *=* *.085). This contrasting result should be treated with caution though, as the elimination of repeated genets significantly reduces the number of replicates and therefore of statistical power. Additionally, the fact that our results followed the same pattern after performing this analysis would indicate that the effect of overrepresentation of a few genets in the sample is quite small. Nevertheless, the identification of the different number of genets present in a sample could be highly important for genetic analysis and for the assessment of ecological effects of genetic diversity in clonal organisms and should be routinely performed (Arnaud‐Haond & Belkhir, 2007; Arnaud‐Haond et al., 2005; Halkett, Simon, & Balloux, 2005).

### 4.4 On the relationship between heterozygosity and phenotypic plasticity

Discussions on the relationship between phenotypic plasticity and heterozygosity as well as on the mechanisms underlying the advantages of heterozygotes over homozygote organisms in terms of their supposedly higher plasticity have, by and large, failed to reach a consensus. We believe this to be due in part to the many different connotations and uses of the concept of phenotypic plasticity (Forsman, 2015) which can give rise to confusion and also to a failure to recognize that genetic diversity is a hierarchical concept which can be applied to different ecological levels (Engelhardt, Lloyd, & Neel, 2014). For example, Schlichting (1986) argues that “Genetic variability is a characteristic of the group, and plasticity is a characteristic of the individual,” to which he adds, “There is no apparent reason why the presence of genetic variability in a population should oppose the evolution of appropriate plastic responses.” We agree with the latter, but we think genetic variability may be a characteristic of both individuals and of populations as described by Engelhardt et al. (2014). Therefore, when genetic variability is measured at the individual level, as is the case with heterozygosity (despite the index being defined for a population), there is actually no apparent reason why it could not be related to plasticity (or to any other individual property). So, how could such a relationship arise?

One of the existent hypotheses that would explain this relationship is the intermediacy of the heterozygote (Gillespie & Langley, 1974). It states that the fitness of genotypes varies over the course of life events or across different environments, but heterozygotes are always intermediate compared to homozygotes, which are specialized for maximizing fitness during a particular event through ontogeny or under particular environmental conditions. It follows that if homozygote and heterozygote organisms are exposed to different environments during their lifespan, heterozygotes will have an advantage and will undergo a relative increase in abundance (Mitton & Grant, 1984). This would explain the association between heterozygosity and phenotypic plasticity and implies that a population with higher heterozygosity would be more advantageous under variable environmental conditions.

Additionally, a negative correlation has been shown to exist between oxygen consumption in marine invertebrates and the number of heterozygous loci within an individual, especially under stressful conditions (Singh & Zouros, 1978; Zouros, Singh, & Miles, 1980). In other words, individuals with higher heterozygosity may be more efficient in their energy use and would require less energy to maintain fitness, leaving them with a higher energy surplus to deal with stressful conditions (Mitton & Grant, 1984). The generality of the above, its applicability in seaweeds, and its underlying mechanisms remain unclear and have yet to be studied. Nevertheless, higher heterozygosity implies more complex genetic machinery and regulatory processes, which may translate to a greater specialization of functions and thus, higher efficiency (Smith, 2007), which would also explain the positive association between heterozygosity and adaptive phenotypic plasticity.

The above are some of the ideas about how heterozygosity could increase an organism fitness and how the relationship between the former and phenotypic plasticity could emerge. However, further research is required across different species and systems to establish the general pattern and the mechanisms underlying it. In this sense, our experiments in *G. chilensis* constitute one of the first conducted in macroalgae to explore this relationship and they suggest a positive correlation between heterozygosity and adaptive phenotypic plasticity, which supports the original ideas suggested by Mitton and Grant (1984). In spite of this, we believe that more experiments and better tools are required to confirm the above conclusion in this, as well as in other marine species. We hope this study encourages other researchers in this matter, as we consider it to be crucial for a better understanding of how organisms can cope with changing environments. This could be especially important in the face of climate change, which is challenging the survival of natural populations worldwide (Hoegh‐Guldberg & Bruno, 2010; Smith, Edmonds, Hartin, Mundra, & Calvin, 2015) and to which plasticity could play a major role (Chevin, Gallet, Gomulkiewicz, Holt, & Fellous, 2013; Lande, 2009; Reusch, 2013).

## CONFLICT OF INTEREST

None declared.

## AUTHOR CONTRIBUTIONS

CGS performed experimental design, experimental execution, data analysis, manuscript preparation, and revision. AG performed experimental design, data analysis, manuscript preparation, and revision. JB and VF performed experimental execution and manuscript revision. BS performed experimental design, experimental execution, manuscript preparation, and revision.
